# 3DVizSNP: a tool for rapidly visualizing missense mutations identified in high throughput experiments in iCn3D

**DOI:** 10.1186/s12859-023-05370-5

**Published:** 2023-06-09

**Authors:** Michael Sierk, Shashikala Ratnayake, Manoj M. Wagle, Ben Chen, Brian Park, Jiyao Wang, Philippe Youkharibache, Daoud Meerzaman

**Affiliations:** 1 Computational Genomics and Bioinformatics Branch, Center for Biomedical Informatics and Information Technology, National Cancer Institute, NIH, Rockville, MD 20852 USA; 2 Faculty of Pharmacy, University of Grenoble Alpes, Grenoble, France; 3 Department of Bioinformatics, Manipal School of Life Sciences, Manipal Academy of Higher Education, Manipal, 576104 India; 4 Digital Services and Solutions Branch, Center for Biomedical Informatics and Information Technology, National Cancer Institute, NIH, Rockville, MD 20852 USA; 5 National Center for Biotechnology Information, National Library of Medicine, NIH, Bethesda, MD 20894 USA; 6 Cancer Data Science Laboratory, Center for Cancer Research, National Cancer Institute, NIH, Bethesda, MD 20892 USA; 7 Present Address: School of Mathematics and Statistics, Faculty of Science, and Computational Systems Biology Group, Children’s Medical Research Institute, University of Sydney, Camperdown, NSW Australia

**Keywords:** Protein structure, Structural bioinformatics, Genomic variation, Single nucleotide polymorphisms, Mutation prioritization, Phenotype impact

## Abstract

**Background:**

High throughput experiments in cancer and other areas of genomic research identify large numbers of sequence variants that need to be evaluated for phenotypic impact. While many tools exist to score the likely impact of single nucleotide polymorphisms (SNPs) based on sequence alone, the three-dimensional structural environment is essential for understanding the biological impact of a nonsynonymous mutation.

**Results:**

We present a program, 3DVizSNP, that enables the rapid visualization of nonsynonymous missense mutations extracted from a variant caller format file using the web-based iCn3D visualization platform. The program, written in Python, leverages REST APIs and can be run locally without installing any other software or databases, or from a webserver hosted by the National Cancer Institute. It automatically selects the appropriate experimental structure from the Protein Data Bank, if available, or the predicted structure from the AlphaFold database, enabling users to rapidly screen SNPs based on their local structural environment. 3DVizSNP leverages iCn3D annotations and its structural analysis functions to assess changes in structural contacts associated with mutations.

**Conclusions:**

This tool enables researchers to efficiently make use of 3D structural information to prioritize mutations for further computational and experimental impact assessment. The program is available as a webserver at https://analysistools.cancer.gov/3dvizsnp or as a standalone python program at https://github.com/CBIIT-CGBB/3DVizSNP.

**Supplementary Information:**

The online version contains supplementary material available at 10.1186/s12859-023-05370-5.

## Background

**Fig. 1:**
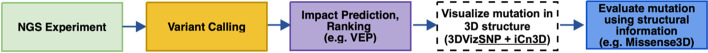
Illustration depicting the role 3DVizSNP fills in the process of evaluating missense mutations. Many programs provide impact predictions for large numbers of SNPs based on sequence (purple box), and there are algorithms that assess the impact of individual mutations using 3D structure (blue box), but 3DVizSNP allows the user to easily visualize a moderate number of mutations in 3D to facilitate prioritization of mutations for further study

High-throughput sequencing experiments generate large numbers of variant calls. Though there are many types of variants, here we focus on nonsynonymous missense mutations affecting protein coding regions. There are databases with annotations of known SNPs, such as dbSNP [1], ClinVar [2], and COSMIC [3], but the volume of data generated by ongoing experiments means that researchers need tools to evaluate the effects of SNPs that, for most, are not present in these databases. The computational challenge is in predicting which variants may impact biological function [4–9]. There are numerous tools available that predict the possible deleterious impact of a mutation, most of which use one-dimensional sequence only [8, 10, 11]. The three-dimensional structural context of variants provides critical information for assessing the potential impact of an amino acid mutation. While the number of experimentally solved 3D structures is still relatively small compared to the number of sequences, the success of deep-learning based structure predictors such as AlphaFold [12] and others [13, 14] have greatly increased the number of proteins with high-quality structural information. The challenge is to make use of all this information in efficient ways. It is typically cumbersome and time consuming for non-structural biologists to identify the protein where a given mutation occurs, determine if the mutation is in a known PDB structure or not, load the PDB structure (if available) or a predicted model, bring up the mutation in a 3D viewer where one can visualize the local structural context for the mutation, and analyze differences due to a mutation in structural terms. Some tools make use of 3D information to predict the effect of a mutation, such as Missense3D [15], HOPE [16], or StructMAn [17], but these tools are generally not set up to operate efficiently on a large number of mutations. They also may require more advanced structural biology and biophysical knowledge to operate and/or interpret the results, while non-structural biologists need to quickly and efficiently make use of the available 3D structural information to help assess the likely impact of a nonsynonymous mutation. Below we describe a tool called 3DVizSNP designed to fill this gap (Fig. 1).

## Implementation

3DVizSNP is a Python program that, combined with the iCn3D structure viewing and analysis platform [18, 19], allows the user to quickly process a VCF file and produce a table with Ensembl Gene IDs, Gene symbols, UniProt IDs, PDB IDs (if applicable), SIFT [20] and PolyPhen [21] predictions and scores, and a link that can open iCn3D highlighting the mutations mapped in 3D on either the relevant PDB structure or the AlphaFold predicted structure. The user can quickly go through tens to hundreds of nonsynonymous mutations identified in high-throughput experiments and visualize the structure and structural contacts to inform and prioritize further studies, without requiring extensive structural biology expertise or heavy computational resources, and without installing a standalone 3D structure viewer or modeling software. It leverages REST APIs including that of iCn3D, which enables the rapid retrieval of experimental structures from the PDB or predictions from the AlphaFold database, and display of contacts in 1D/2D/3D formats, all through a saveable and shareable URL [18].

### 3DVizSNP workflow

**Fig. 2:**
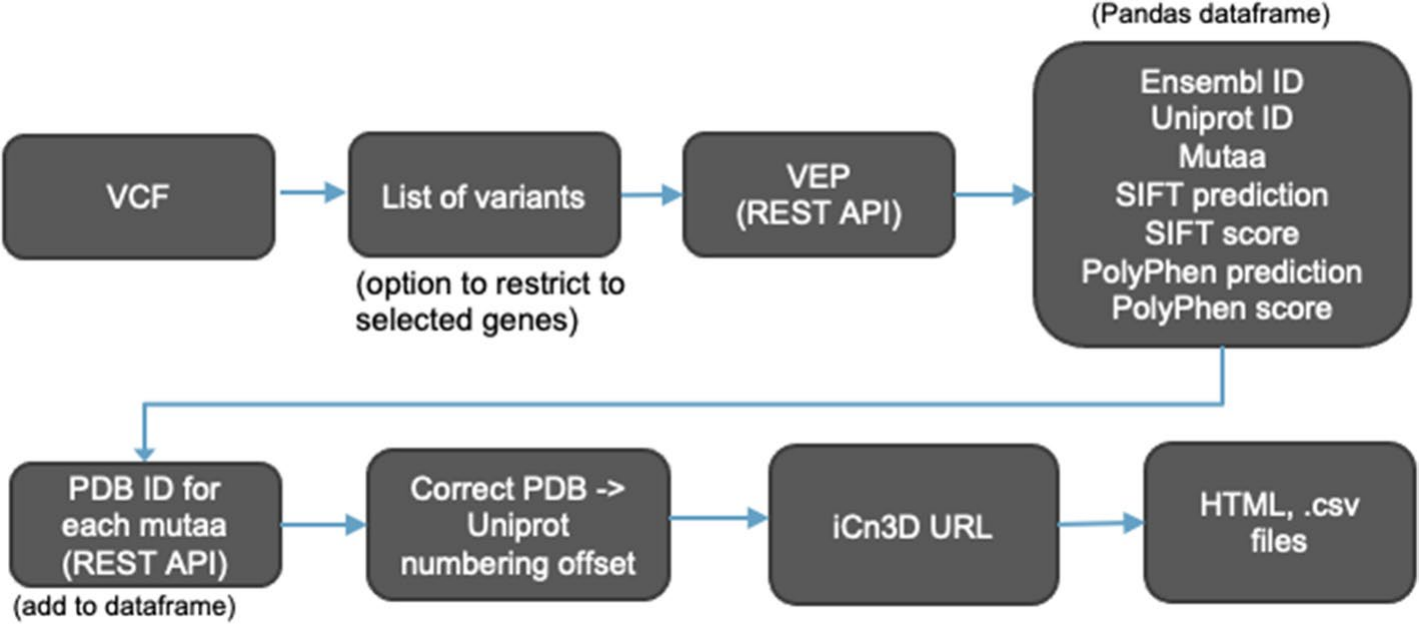
The 3DVizSNP Workflow

3DVizSNP reads in a bgzipped VCF file with an associated tabix index and extracts the variants, with the option to only select variants from an input list of HGNC gene symbols. It then submits the variants to the VEP server [11] via a REST API and gets back the Ensembl Gene IDs, Gene symbols, SwissProt IDs, amino acid mutation, and SIFT and PolyPhen predictions and scores. It uses the PDB REST API to identify the PDB ID of the X-ray crystal or electron micrograph with the highest resolution, or the NMR structure in the absence of X-ray or EM structures, for each amino acid mutation (correcting for mismatches between UniProt and PDB numbering). It also avoids structures with engineered mutations at the mutation site. If an experimental structure is not available, it loads the AlphaFold prediction (for sequences under 2700 amino acids in length) color-coded by prediction confidence. The output is an HTML table and a comma separated value (csv) file, which includes iCn3D links for each mutation. (Fig. 2) The links can be clicked to open the PDB structure or AlphaFold predicted structure in iCn3D, with the mutations added as separate tracks in the 1D viewer for SIFT and PolyPhen predictions. The iCn3D ‘scap interactions’ command is used to highlight the wild-type and mutant amino acid side chains (which can be toggled) in iCn3D. iCn3D has the capability to add multiple annotations to the sequence viewer, enabling the user to quickly determine if the variant is in a known functional domain, matches known mutations in dbSNP or ClinVar, etc. The csv file can be loaded into a spreadsheet program, enabling the user to record notes about variants, sort them, store the results, etc.

**Fig. 3:**
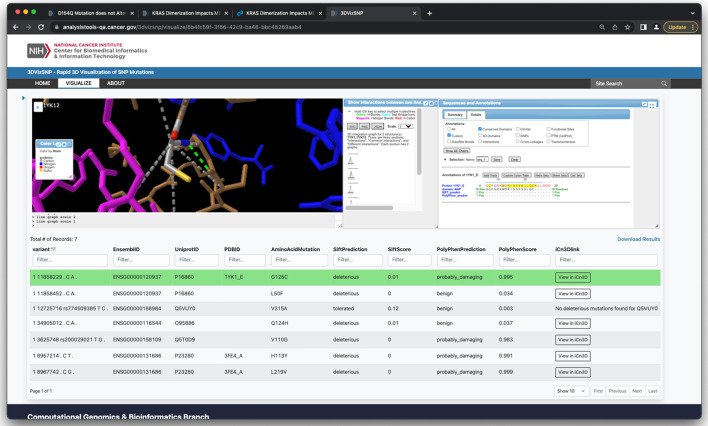
The web interface to 3DVizSNP. The current row is highlighted in green and shown in the embedded iCn3D interface. Clicking on a different row loads that mutation in the iCn3D viewer. The table is sortable and filterable, and clicking on the ‘View in iCn3D’ button opens the mutation in a new full page iCn3D

In addition to the python script, we have provided a website where the user can upload a VCF file and view the sortable, filterable output table along with an embedded version of iCn3D (Fig. 3). Clicking on a row brings up the mutation in iCn3D above the table, while clicking on the iCn3D button will open the mutation in a separate window with the full version of iCn3D.

## Results

### Known KRAS mutations

**Fig. 4:**
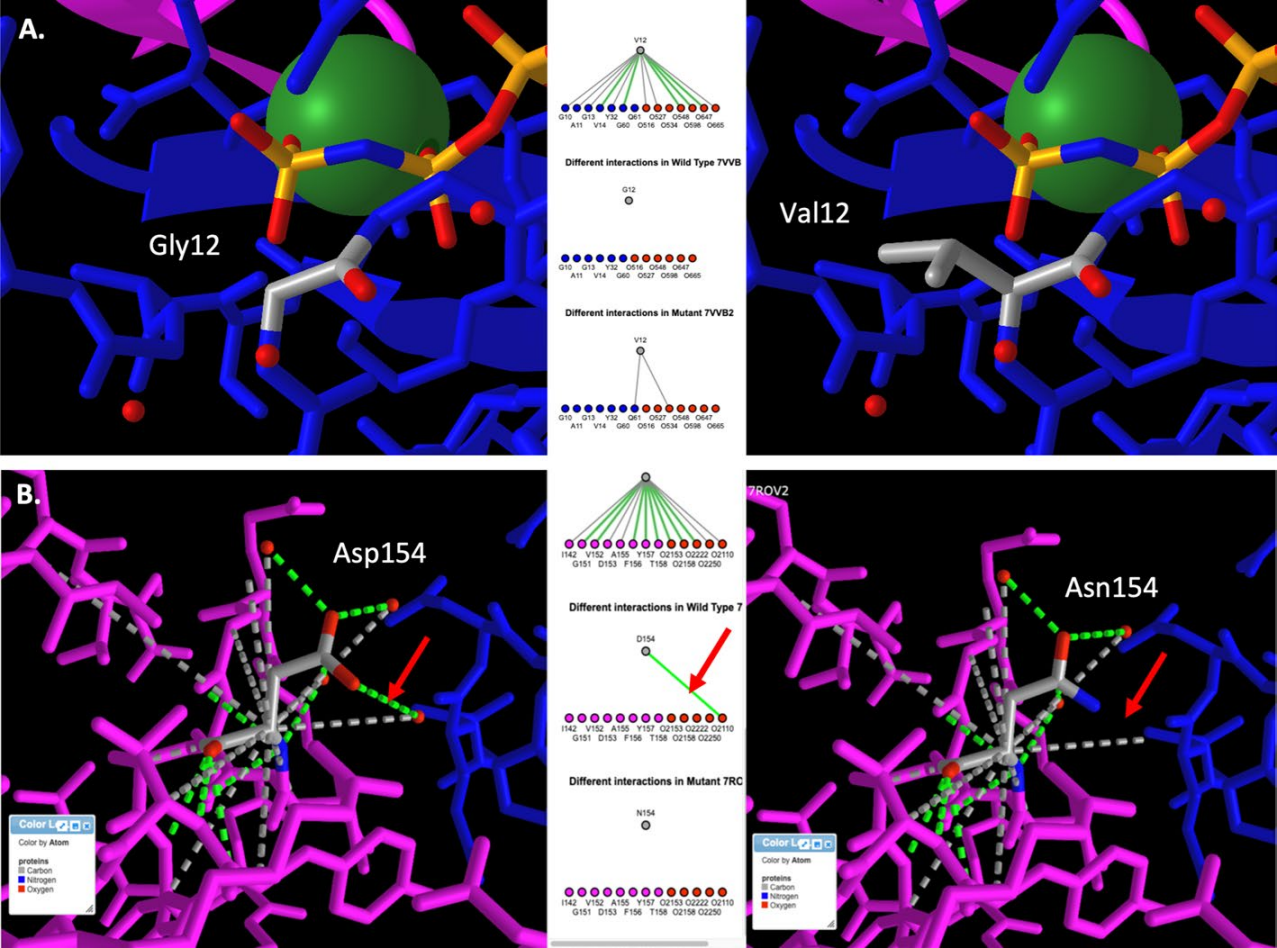
Mutations in the KRAS protein. **A** The G12V mutation (WT, left; G12V, right). The SIFT score is zero and the prediction is ‘deleterious_low_confidence’. The active site magnesium ion (green sphere) and nonhydrolyzable ATP analog GNP are seen near the mutated residue. **B** The D154N mutation (WT, left; D154N, right). The SIFT score is zero and the prediction is ‘deleterious_low_confidence’. The red arrows highlight the hydrogen bond to a water molecule that is removed by the mutation

To illustrate the ability to find and visualize known mutations in the KRAS oncogene, we ran 3DVizSNP against a colorectal adenocarcinoma (COAD) vcf file from a single patient from the TCGA database with the flag ‘-g KRAS’ to select only variants in the KRAS gene. This produced an output table with two mutations, the well-known G12V mutation and a 2nd mutation, D154N. Figure 4 depicts these mutations in iCn3D. The G12V mutation is predicted to be ‘deleterious_low_confidence’ by SIFT (with a score of 0) and is not called in this instance by PolyPhen. (Note that the PDB ID 7rov has the lowest resolution structure, so it is used for the D154N mutation, but it is an engineered G12D mutant, so 3DVizSNP discards it in favor of 7vvb for the G12V mutation.) The impact of the G12V mutant is not obvious upon initial inspection, but it is adjacent to the GTP binding site and involves replacing a glycine in a turn with a valine, which is likely to cause steric hindrances. (Fig. 4A) The D154N mutation is also predicted to be ‘deleterious_low_confidence’ by SIFT with a score of 0 and is not called by PolyPhen. The mutation is subtle, only appearing to disrupt a single hydrogen bond to a water molecule (Fig. 4B). However, KRAS activity depends upon dimerization and D154 helps stabilize the alpha-alpha form of the KRAS dimer [22]. It has been reported that a D154Q mutation affects dimerization and has a growth inhibitory effect on oncogenic versions of KRAS, [23] though the impact of mutating this residue on dimerization is disputed [24].

### TCGA VCF example

**Fig. 5:**
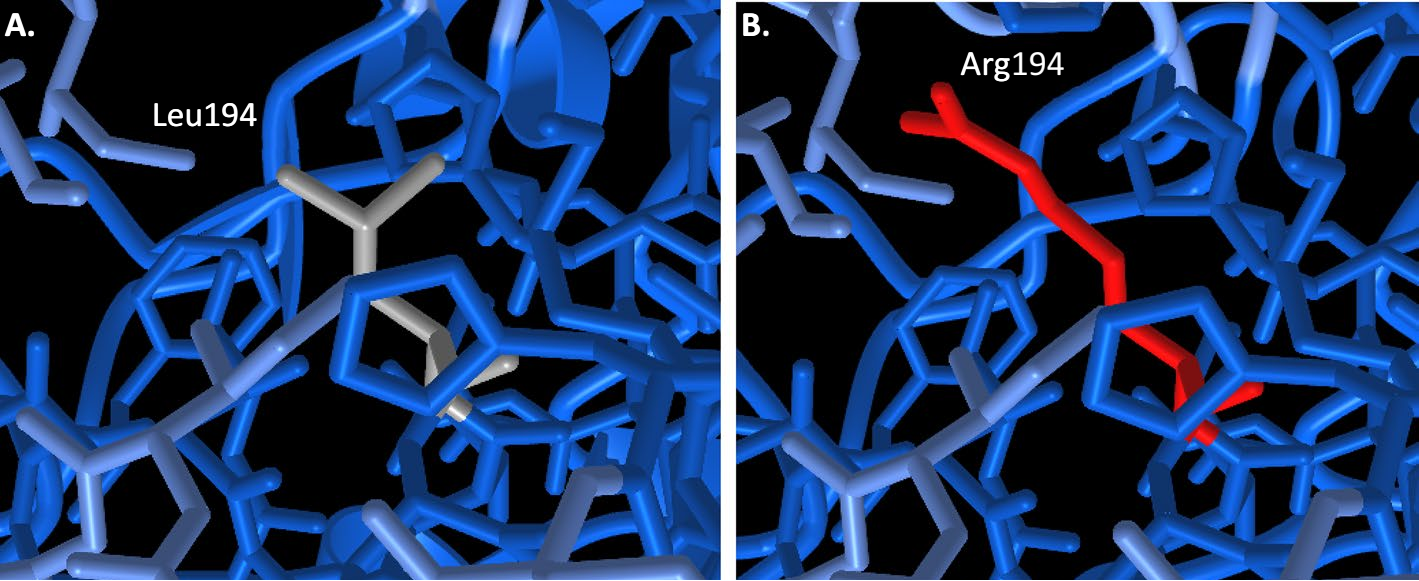
A closeup view of the wildtype leucine 194 (gray) and the mutation to arginine (red) in the AlphaFold prediction for UniProt ID Q5TCH4, a Cytochrome P450 4A protein. The mutation was identified in a vcf file from TCGA (see text for details). The blue color of the other residues indicates that this is a very high confidence region for the AlphaFold structure prediction

To illustrate the utility of 3DVizSNP in screening mutations of unknown effect, we took a randomly selected VCF file from a Glioblastoma patient in the TCGA database, which had 27,526 variants in it. We submitted it to the OpenCRAVAT server [10] to filter the list down to a more manageable size, by using the filters coding = yes, polyphen-2 HumVar rank score 0.8, and SIFT prediction ‘damaging’. This produced a list of 901 variants, which we ran through 3DVizSNP. We looked at each variant in iCn3D and investigated variants that met certain criteria for further inspection. These criteria included being in a PDB structure or in a Confident or Very High region of an AlphaFold prediction, not being on the surface of the structure, and having significant changes in the structure, such as changes in size/polarity/charge, changes in contacts, etc. Low confidence areas of AlphaFold predictions are typically not well-ordered and little inference can be drawn regarding the effects of mutations in those regions. We avoided surface mutations because it is harder to predict what effects they might have on protein structure and function. Obviously, they can affect binding to other proteins but in the absence of knowledge about these interfaces one cannot make quick determinations of the impact of mutations on binding. We then looked at the UniProt page for information on the protein function, known mutations, etc. We also submitted the mutation to the Missense3D server [15] and the SAAMBE server [25] to obtain structure-based predictions of mutational impact. The 44th mutation in the output list was a L194R mutation in UniProt ID Q5TCH4, a Cytochrome P450 4A protein. (Fig. 5) The Missense3D server predicts it causes structural damage, as it introduces a buried hydrophilic and charged residue for a buried hydrophobic residue. The SAAMBE server predicts the mutation is disruptive with a ΔΔG of 0.31 kcal/mol (that is, the free energy of folding of the mutant protein is predicted to be 0.31 kcal/mol higher than that of the wild-type). The UniProt entry has a link to the DisGenNET database (entry 284541) which links mutations to diseases. DisGenNET lists liver carcinoma as a disease linked to CYP4A22, and links to PubMed ID 30069903 [26], which indicates that higher CYP4A22 expression in hepatocellular carcinoma correlates with better disease prognosis. Obviously hepatocellular carcinoma differs from glioblastoma; the point is not to make a strong claim that this particular mutation contributes to glioblastoma progression, but rather to point out that there is evidence linking mutations that damage CYP4A22 function, as the L194R mutation is predicted to, to cancer progression, indicating further study is warranted. This process illustrates that one can use tools such as OpenCRAVAT to filter a larger set of variants, then use 3DVizSNP to further sort and prioritize variants based on the available 3D structural information. One can then use other tools and databases such as Missense3D, SAAMBE, and UniProt to derive further support for the potential functional relevance of a given mutation. Continuing this process could winnow down the list of variants to around 50 for further in-depth computational assessment and experimental characterization.

## Discussion

There are other tools that allow one to visualize missense mutations in a 3D context. (Additional file 1) PhyreRisk from the Sternberg lab does similar things to 3DVizSNP [27]. It accepts more formats for submitting variants than 3DVizSNP, also uses the VEP server, displays the list of variants in a table, and provides an interactive 3D interface for visualizing the mutation. The main advantages of 3DVizSNP are the ability to toggle between the mutant and wild-type residues, the depiction of intramolecular contacts for the wild-type and mutant, the use of Alphafold models instead of Phyre models, and the fact that the structure viewer is embedded in the webpage with the output table. Phyre has been used for many years to successfully model protein structures, but it does not have the same accuracy or breadth of coverage that AlphaFold2 does [28]. MuPit from the Karchin lab also displays mutations on 3D structures along with annotations [29, 30]. Like PhyreRisk, it does not allow toggling between the mutant and wild-type residue and does not show intramolecular contacts, and it is limited to mapping mutations onto PDB structures only. VIVID is a recently developed tool designed to accomplish many of the same tasks as 3DVizSNP [31]. It requires the user to input a gene sequence, a VCF file with mutations, a GFF file, and a PDB file (which can be obtained from an Alphafold prediction). It allows visualization of nonsynonymous mutations on a 3D structure, along with a variety of metrics including both population level information and mutational impact calculations. It appears to be limited to evaluating one protein structure at a time, and does not allow toggling between the wild-type and mutant sidechains.

3DVizSNP provides complementary features to these existing powerful tools. Generally speaking, these other tools are designed from the perspective of viewing multiple mutations on a particular protein structure, whereas 3DVizSNP is designed to from the perspective of viewing mutations from a particular VCF file. The existing tools provide less ability to screen large numbers of mutations found in a VCF file than 3DVizSNP while providing more in-depth information about each mutation or protein.

While 3DVizSNP provides significant novel capabilities, it does have limitations. Due to querying REST APIs it is not particularly fast. It takes 3 min, 21 s to produce output for 1000 variants on a MacBook Pro laptop with 2.3 GHz 8-Core Intel Core i9 processor and can take closer to 30 min for longer input VCF files. Nonetheless the running time is small compared to the time required to visually assess tens to hundreds of mutations, and is comparable to the running times of the servers discussed above. 3DVizSNP requires filtering of VCF files (which may have millions of variants) in advance using tools like vcftools [32], bcftools [33], or OpenCRAVAT [10]), as it is not practical to use with more than approximately 1000 variants in the output.

## Conclusions

Here we present a tool, 3DVizSNP, that facilitates the rapid assessment of phenotypic impact of missense mutations extracted from VCF files by enabling the user to visualize multiple mutations quickly in the iCn3D web-based structure and sequence analysis platform. This tool provides structural context not present in sequence-based impact prediction programs but enables the viewing of multiple mutations more rapidly and easily than other 3D impact assessment programs. This tool will make it easier for researchers to prioritize mutations for further study, a critical bottleneck in modern high-throughput experiments.

## Availability and requirements

Project name: 3DVizSNP. Project home page: https://analysistools.cancer.gov/3dvizsnp. (Source code: https://github.com/CBIIT-CGBB/3DVizSNP). Operating system(s): Platform independent. Programming language: Python. Other requirements: Pysam v. 0.19.1, Pandas library. License: MIT License.

## Supplementary Information

**Additional file 1.** A table summarizing the features of 3DVizSNP, PhyreRisk, MuPit, and VIVID.

## Data Availability

The python code presented here is publicly available at https://github.com/CBIIT-CGBB/3DVizSNP. The webserver is publicly available at https://analysistools.cancer.gov/3dvizsnp. Example VCF files used are publicly available at the Github, at TCGA, or from the authors upon request.

## Abbreviations

SNP: Single nucleotide polymorphism
PDB: Protein data bank
VCF: Variant caller format
REST: Representational state transfer
API: Application programming interface
